# Dissociable functional roles of the human action-observation network (Commentary on E. S. Cross *et al.*)

**DOI:** 10.1111/j.1460-9568.2009.06958.x

**Published:** 2009-10

**Authors:** James M Kilner

**Affiliations:** The Wellcome Trust Centre for Neuroimaging, IoNUCL, 12 Queen Square, London WC1N 3BG, UK

When we observe other people executing an action we are able to estimate what the underlying intentions of the person performing the action are. However, little is known about the neural mechanisms underlying this ability. Many believe that this ability is made possible by responses in a network of brain areas that are consistently reported to be active when observing actions. This action-observation network (AON) consists of three bilateral cortical areas that are reciprocally connected, the ventral premotor cortex, inferior parietal lobule and superior temporal sulcus. (As some of the areas of the AON contain mirror neurons (Rizzolatti & Craighero, 2004; Chong *et al.*, 2008; Kilner *et al.*, 2009) this network is sometimes referred to as the mirror neuron system). Despite over a decade of neuroimaging research on the AON little is known about the functional role of each of these areas of the AON when observing actions.

In this issue of *EJN*, Cross *et al.* (2009) investigated dissociable functional roles of different areas of the human AON by using a dance-learning paradigm. Subjects were trained prior to scanning to learn different sequences of a computer dance game. In the game subjects watched a scrolling display of moving arrows, which cued the direction of the dance step to make. Subjects learned dance sequences that were indicated by the arrows alone and also sequences where videos of a human expert dancer were superimposed on the scrolling arrows. Cross *et al.* (2009) then recorded the BOLD signal whilst subjects observed videos that showed sequences of dance cues that were either trained or untrained and in which there was a human dancing or not. The results showed that the bilateral superior temporal cortex responded preferentially to videos with a human present, regardless of training experience, whereas the right ventral premotor cortex responded more strongly when observing sequences on which they had been trained, regardless of whether a human was present or not. The authors argue that this demonstrates that different areas of the AON show different functional properties, the superior temporal cortex differentiating the form of the observed movement, and the right ventral premotor cortex response depending upon subjects’ prior experience of the action irrespective of the form.

The result for the right ventral premotor cortex is interesting for at least two reasons. First, it demonstrates that areas of the AON are active even when the subjects are not observing a human form. The key factor appears to be the subjects’ prior experience of associating the visual cues to actions in the training phase irrespective of the whether the observed cue contained a human performing an action or not. This result is consistent with recent theoretical accounts of why the human motor system is active when observing actions (Brass & Heyes, 2005; Kilner *et al.*, 2007). According to these accounts, the AON is active during observation of an action because the cause of the action and the visual and proprioceptive visual expression or effect of that action are mapped through changes in connection strengths during periods of perceptual or associative learning. The second reason why it is interesting is that this pattern of activity was present only in the right ventral premotor cortex and no similar pattern was observed in the corresponding region of the left ventral premotor cortex. This suggests that right and left ventral premotor cortex have dissociable functional roles when observing actions. However, it is not clear what these different roles are. A critical next step will be to establish why the AON is active when subjects are observing actions and what are the different functional roles for each of the areas of the AON.

## References

[b1] Brass M, Heyes C (2005). Imitation: is cognitive neuroscience solving the correspondence problem?. Trends Cogn. Sci..

[b2] Chong TT, Cunnington R, Williams MA, Kanwisher N, Mattingley JB (2008). fMRI adaptation reveals mirror neurons in human inferior parietal cortex. Curr. Biol..

[b3] Cross ES, Hamilton AFdC, Kraemer DJM, Kelley WM, Grafton ST (2009). Dissociable substrates for body motion and physical experience in the human action-observation network. Eur. J. Neurosci..

[b4] Kilner JM, Friston KJ, Frith CD (2007). The mirror-neuron system: a Bayesian perspective. Neuroreport.

[b5] Kilner JM, Neal A, Weiskopf N, Friston KJ, Frith CD (2009). Evidence of mirror neurons in human inferior frontal gyrus. J. Neurosci..

[b6] Rizzolatti G, Craighero L (2004). The mirror-neuron system. Annu. Rev. Neurosci..

